# On Covalent N_2_H_6_: A High-Energy, Non-Lewis, Local Minimum That May (Not) Exist

**DOI:** 10.1021/acs.jpca.6c01311

**Published:** 2026-04-06

**Authors:** Kelling J. Donald, George Ruthven

**Affiliations:** Department of Chemistry, Gottwald Center for the Sciences, 6888 University of Richmond, Richmond, Virginia 23173, United States

## Abstract

Saturated molecules of period 2 *p*-block atoms, such as CH_4_, NH_3_, and H_2_O with full octets, are not expected to dimerize by covalent bond formation. A computational examination of NH_3_ as an exception is carried out. Quite high in energy relative to the isolated monomers, the covalently bound local singlet hexahydridodinitrogen (N_2_H_6_) minimum of *D*_*3d*_ symmetry on the potential energy surface of the ammonia pair is probed. The structure and bonding of this double-octet dimer system are confirmed, but we find that the location of the species on the potential energy surface is highly sensitive to model chemistry, especially for post-Hartree–Fock methods. Of several singlet dimer pairs examined herein, no other system with period two *p*-block central atoms formed stable covalent minima. The prospect for dissociation through competing channels provides strong reasons for pessimism about any realization of the N_2_H_6_ species. Yet, the observed energy lowering for N_2_H_6_, which is strengthened by Rydberg bonding, challenges chemists to continue pressing against the perceived limits of chemical bonding.

## Graphical abstract

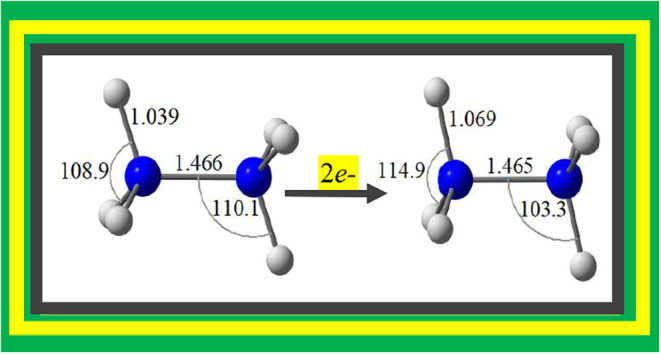

## Introduction

The capacity for and limits of covalent bonding by periods 1 and 2 main group atoms are thought to be quite well established. A standard narrative has long been codified in modern textbooks;^1,2^ it relies on the deployment of so-called Lewis structures—a sublime approach rooted in a ground-breaking paper published over a hundred years ago^3^ with subsequent contributions form Langmuir and others,^4–8^ —and bolstered later on by the valence shell electron pair repulsion (VSEPR) model.^9–11^

The octet rule and the two-center two-electron (*2-c, 2-e*) bond are cornerstones of that basic bonding picture and compounds limited to elements in and above period 2 (dubbed “period 2 compounds” in this work) are expected to be its most faithful adherents. Yet chemical bonding in period 2 compounds abound with decisive features beyond the simple *2-c*, *2-e* picture, such as multicenter bonding (diborane), aromaticity (benzene), hyperconjugation (ethane), intramolecular hydrogen bonding (acetylacetone) and more. Indeed, the unique combination of size and orbital effects in period 2 makes some of those features—such as π-delocalization and aromaticity—most prominent in that region of the periodic table. And the exceptional atomic properties of period 2 atoms can show up in remarkable ways even in the physicochemical properties of small molecules such as the net C– ← O+ direction of the dipole moment of CO^12–14^ and the weakness of the F_2_ bond.^15–18^

Nonetheless, boundaries still persist for the chemical bonding in period 2 compounds. Hypervalence, for example, remains off limits for period 2 central atoms. For *p*-block central atoms below period 2, e.g., Si, P, S, and Cl, where compounds such as SiF_4_(NH_3_)_2_, PF_5_, SF_6_, and ClF_3_ fail to surprise even undergraduate chemistry majors, hypervalence can hardly even be cast as an exception. But the experimental preparation of any bottleable C, N, O, or F analogue, respectively, of those Si, P, S, and Cl compounds would astonish. And that is so despite debates about the meaning and relevance of hypervalence as a concept in modern chemistry,^19–25^ and efforts to propose and prepare hypercoordinate period 2 compounds.^26–28^

In this contribution, we consider computationally the high energy N_2_H_6_ system^29^ that brushes up we think against the assumed limits of chemical bonding in period 2 compounds. A still experimentally unprecedented covalent unit, it may be seen—by one view—as the outcome of a redistribution of electron density between two stable saturated period 2 molecules: H_3_N-NH_3_. Several substituted analogues of this system that we considered exhibited no covalent energy lowering, exposing N_2_H_6_ as rather unique among period 2 systems in its ability to form a double octet covalent pair.

So-called pnictogen bonding^30^ involves the weak attractive interaction between a strongly polarized group 15 central atom (a pnictogen, hence the moniker) acting as a Lewis acid and an electron donor of some sort, Y, as in a Cl_3_P←:Y type complex, for instance. The resulting weak dimer is asymmetric, since the lone pair on Y avoids coincidence with the lone pair on P, and is directed instead toward one of three (in the Cl_3_P case) locally positive regions (sigma holes) on the P center—one opposite each P–Cl bond along the extension of that bond. A complex such as F_3_N←NH_3_ would be a pnictogen bonded pair. But N_2_H_6_ considered here is not such a pair at all.

The weakly bound dimer of ammonia have been extensively studied too.^31,32^ That system is held together by weak H_3_N···H-NH_2_ hydrogen bonding and dispersion interactions with a long “N---N” distance in the complex. The highly symmetric (and very high energy) covalently bound system identified here, however, is quite different, exhibiting an extraordinary mode of bonding. This N_2_H_6_ system is also fundamentally different from the purely Rydberg bonded (NH_4_)_2_;^33^ the latter is a weak pairing of two neutral Rydberg radicals,^34,35^ but N_2_H_6_ is a covalent pairing of two neutral octets to form what may be seen as an N–N double σ bond that involves but is not at all solely constituted by Rydberg bonding.

## Computational Methods

All of the structural optimizations reported in this work were carried out using the Gaussian 16 (G16) software^36^ unless otherwise specified. For each optimized structure, the nature of that position on the potential energy surface was established by a harmonic vibrational frequency analysis. The structures reported in this work have been confirmed to be local minima, having no imaginary frequency at the level of theory at which they were optimized. We employed both density functional and *ab initio* approaches for those optimizations, including the HF,^37^ B3LYP,^38^ ωB97XD,^39^ M06-2X,^40^ MP2,^41,42^ MP4,^43,44^ QCISD,^45^ QCISD­(T)^45–47^ CCSD,^48,49^ and CCSD­(T)^45,48–50^ methods, in combination with both cc-pVTZ and aug-cc-pVTZ type basis sets.^51,52^ Inclusion of the Hartree–Fock (HF) method allows us by direct contrast with other *ab initio* methods to assess the influence of correlation on bonding. Additionally, we combined separately the ωB97XD and CCSD­(T) methods with a number of different basis sets—6-311+G*,^53–55^ 6-311++G**,^53–55^ def2-TZVPP,^56,57^ def2-QZVPP,^56,57^ cc-pVDZ,^58,59^ cc-pVTZ,^51,59^ cc-pVQZ,^52,59^ and cc-pV5Z^59,60^ —to probe further the basis set dependence of our observations.

Molecular orbital representations discussed in this work were generated using the GaussView 6 program^61^ and simple ball and stick molecular structures were produced using the Chemcraft graphical user interface.^62^ The Multiwfn software^63,64^ is used to carry out an Adaptive Natural Density Partitioning (AdNDP) analysis (based on a computational Natural Bond Orbital analysis carried out using the G16 suite of programs) as part of a detailed assessment of the bonding in the main species considered in this work. The Amsterdam Density Functional (ADF 2022.990) software is employed in one instance in our investigation for structural optimization as well.^65,66^

## Results and Discussion

The optimization of the ammonia dimer at an unreasonably short intermonomer separation, carried out for completeness as part of an investigation of weak dimers, returned the covalently bound N_2_H_6_ unit that appears to run counter to certain conventional expectations about chemical bonding. The outcomes of that optimization using both the ωB97XD and coupled cluster CCSD­(T) methods with an augmented triple-ζ Dunning-type basis set for all atoms is shown in Figure 1.

**Figure 1:**
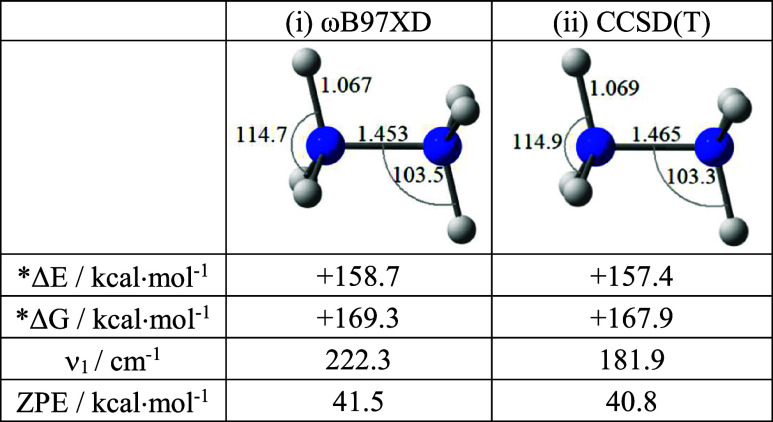
Optimized N_2_H_6_ structures obtained at the (i) ωB97XD/aug-cc-pVTZ and (ii) CCSD­(T)/aug-cc-pVTZ levels of theory. For comparison, the corresponding N–H distance and H–N–H bond angle in ammonia are 1.011 Å and 107.3°, and 1.015 Å and 106.4°, respectively. *The relative energy, Δ*E* = *E*(N_2_H_6_) – 2 × *E*(NH_3_), and similarly for Δ*G*. ZPE ≡ Zero-point energy.

The resulting hexahydridodinitrogen N_2_H_6_ system^29,67^ is very high in energy (see Figure 1), but it is also—considering at least the short N–N distance—rather remarkable for its structural features. Judging at this point from the geometry alone, the N_2_H_6_ unit is covalently bound, with an N–N bond between the two saturated NH_3_ centers (see Figure 1) within just 0.03 Å of the N–N distance (1.424 Å (wB97XD); 1.445 Å (CCSD­(T))) in hydrazine! Like HF, H_2_O, and CH_4_, which have complete octets around the period 2 atom (M), NH_3_ is not expected to possess a covalent minimum on the dimer surface, not at least without some form of coercion (such as high pressure). The H-mediated 3-center 2-electron bridges that facilitate dimerization for hypovalent BH_3_, are well-known, but neither H_n_M-MH_n_ nor H-bridged type dimers have been identified experimentally for the C, N, O, or F compounds just mentioned, though H_3_N-NH_3_ has been discussed in the context of bonding between Rydberg electrons in heterorganic systems.^29^

Given thus the substantial clash between standard expectations and observations on N_2_H_6_, and the possibility, however remote, that the computed N_2_H_6_ minimum in Figure 1 is an unphysical result, we carried out a series of additional studies to probe the method and basis set dependence of our observations. For completeness for each set of data, some of the aug-cc-pVTZ results mentioned earlier will be echoed in subsequent tables.

### Method Dependence

The outcomes of a series of structural optimizations of the N_2_H_6_ species using ten different methods in tandem with the cc-pVTZ type basis set as well as the larger aug-cc-pVTZ basis sets are shown in Tables 1 and 2, respectively. In each case, the structures have been confirmed to be local minima by harmonic frequency analyses.

**Table 1:** Computed Geometrical and Frequency Data (Lowest Frequency, ν_1_) Obtained from a Study of the Method Dependence of the Properties of N_2_H_6_, Employing cc-pVTZ Basis Sets

	HF	B3LYP	ωB97XD	M06-2X	MP2 (full)	MP4 (full)	QCISD	CCCSD	QCISD(T)	CCSD(T)
	Bond distances (Å)									
N–N	1.430	1.476	1.463	1.458	1.476	N_2_H_4_ + H_2_	1.465	1.464	N_2_H_4_ + H_2_	N_2_H_4_ + H_2_
N–H	1.048	1.086	1.082	1.079	1.084		1.078	1.078		
	Angles (deg)									
N–N–H	104.4	101.0	101.0	102.2	99.7		101.1	101.2		
H–N–H	114.1	116.5	116.4	115.6	117.2		116.4	116.3		
	Lowest vibration frequency, ν_1_ (cm^–1^) (uncorrected)									
ν_1_	283.2	110.0	172.2	235.6	101.6		131.1	133.1		

**Table 2:** Computed Geometrical and Frequency Data (Lowest Frequency, ν_1_) Obtained from a Study of the Method Dependence of the Properties of N_2_H_6_, Employing Aug-cc-pVTZ Basis Sets

	HF	B3LYP	ωB97XD	M06-2X	MP2 (full)	MP4 (full)	QCISD	CCCSD	QCISD(T)	CCSD(T)
	Bond Distances (Å)									
N–N	1.425	1.464	1.453	1.449	1.458	1.463	1.454	1.453	1.466	1.465
N–H	1.034	1.071	1.067	1.064	1.070	1.069	1.062	1.062	1.069	1.069
Angles	Angles (deg)									
N–N–H	106.9	103.4	103.5	104.6	102.6	102.989	104.0	104.0	103.3	103.3
H–N–H	111.9	114.8	114.7	113.9	115.3	115.1	114.3	114.3	114.9	114.9
	Lowest vibration frequency, ν_1_ (cm^–1^) (uncorrected)									
ν_1_	287.3	185.6	222.3	265.4	217.3	201.2	222.1	223.2	211.3	181.9

One of the most revealing features of this analysis is the failure of some of the more demanding *ab initio* approaches—specifically the fourth-order Møller–Plesset MP4­(full) method, quadratic configuration interaction method with single and double excitations and the perturbative inclusion of triples (QCISD­(T)), as well as the coupled cluster alternative CCSD­(T)—to locate and return minimum energy N_2_H_6_ structures with the cc-pVTZ basis set. In those cases, a hydrogen atom is released from each N center to yield a hydrazine (N_2_H_4_) molecule. That outcome suggests that barriers to channels leading to dissociation may be particularly low and that access to such channels may be particularly sensitive to the choice of basis set.

The latter interpretation betrays indeed an additional piece of insight that is afforded by the use of the same basis set augmented with diffuse functions on the H and N centers (aug-cc-pVTZ). In that case, a local minimum is indeed identified and confirmed for all of the nine methods considered in this work (Table 2). Of note, the computed geometrical parameters for the unusual N_2_H_6_ species (especially if we exclude the HF results) show remarkably little variation from one model chemistry to the other.

### Basis Set Dependence

From our analysis so far, it is evident that the model chemistry can be decisive for whether an unconstrained optimization yields the N_2_H_6_ structure or not. We have shown (see Table 1) that, without diffuse functions, hydrazine formation is accomplished when some of the most demanding methods are deployed with cc-pVTZ basis sets. And probing the dependence of our results on the choice of basis sets even further proves to be quite revealing. Repeating the N_2_H_6_ optimization using the ωB97XD density functional method combined with a range of basis set types and sizes (Table 3), an N_2_H_6_ minimum is located in all cases except one. The collapse to hydrazine and H_2_ is observed in that case only for the double-ζ (cc-pVDZ) basis set, which is smaller and less flexible than other basis sets included in Table 3. Yet, at the Hartree–Fock level, an N_2_H_6_ structure was recovered in all eight cases (see the Supporting Information (SI)), providing more evidence that locating the N_2_H_6_ minimum on the (NH_3_)_2_ surface depends significantly on model chemistry.

**Table 3:** Data Showing the Basis Set Dependence of N_2_H_6_ Optimization Using the ωB97XD Method

	6-311+G*	6-311++G**	def2-TZVPP	def2-QZVPP	cc-pVDZ	cc-pVTZ	cc-pVQZ	cc-pV5Z
	Bond Distances (Å)							
N–N	1.450	1.453	1.460	1.456	N_2_H_4_ + H_2_	1.463	1.456	1.454
N–H	1.066	1.070	1.080	1.075		1.082	1.074	1.073
Angles	Angles (deg)							
N–N–H	102.6	103.6	101.4	102.1		101.0	102.1	102.5
H–N–H	115.4	114.6	116.2	115.7		116.4	115.7	115.5

That observation is supported strongly by the data in Table 4, which summarizes our results from a study of the basis set dependence of N_2_H_6_ optimization at the CCSD­(T) level. In that case, the cut off (the point at which an N_2_H_6_ type starting structure collapses to hydrazine and H_2_) appears to arise even earlier as the basis set gets smaller. Starting at the quadruple rather than the quintuple zeta basis set due to computational demand, the CCSD­(T) calculations returned an N_2_H_6_ structure in all cases except for the cc-pVDZ and cc-pVTZ basis sets.

**Table 4:** Data Showing the Basis Set Dependence of N_2_H_6_ Optimization Using the CCSD­(T) Method

	6-311+G*	6-311++G**	Def2TZVPP	Def2QZVPP	cc-pVDZ	cc-pVTZ	cc-pVQZ
	Bond Distances (Å)						
N–N	1.453	1.463	1.475	1.465	N_2_H_4_ + H_2_	N_2_H_4_ + H_2_	1.467
N–H	1.059	1.068	1.082	1.076			1.076
Angles	Angles (deg)						
N–N–H	104.0	104.3	100.8	101.9			101.6
H–N–H	114.3	114.1	116.6	115.9			116.0

Given the unique features of this N_2_H_6_ system, we carried out an additional structural optimization and vibrational frequency analysis using a different level of theory (B3LYP-D3/TZP) as implemented in the Amsterdam Density Functional (ADF) software.^65,66^ Those calculations returned indeed the now familiar *D*_*3d*_ N_2_H_6_ minimum energy structure, with an N–N bond distance of 1.463 Å and N–H distances of 1.075 Å, completely in line qualitatively with the *D*_*3d*_ arrangement identified already at various other levels of theory (Tables 1–4).

### What of the Bonding?

A pressing question in our analysis of this compact minimum on the (NH_3_)_2_ potential energy surface is how to account for the bonding in the system. In confronting that question we examined the frontier molecular orbitals for insight into where the electron density is located in the system and to assess as well the separations between the highest occupied and lowest unoccupied molecular orbitals (the HOMO–LUMO gap) in the N_2_H_6_ species. Given the close agreement between the different levels of theory on the structure of the molecule, we focus in this analysis on results obtained at the ωB97XD/aug-cc-pVTZ level of theory.

The full set of occupied N_2_H_6_ molecular orbitals that arise primarily from the valence orbitals of H and N are shown in Figure 2, with two views provided for clarity on the nature of each MO.

**Figure 2:**
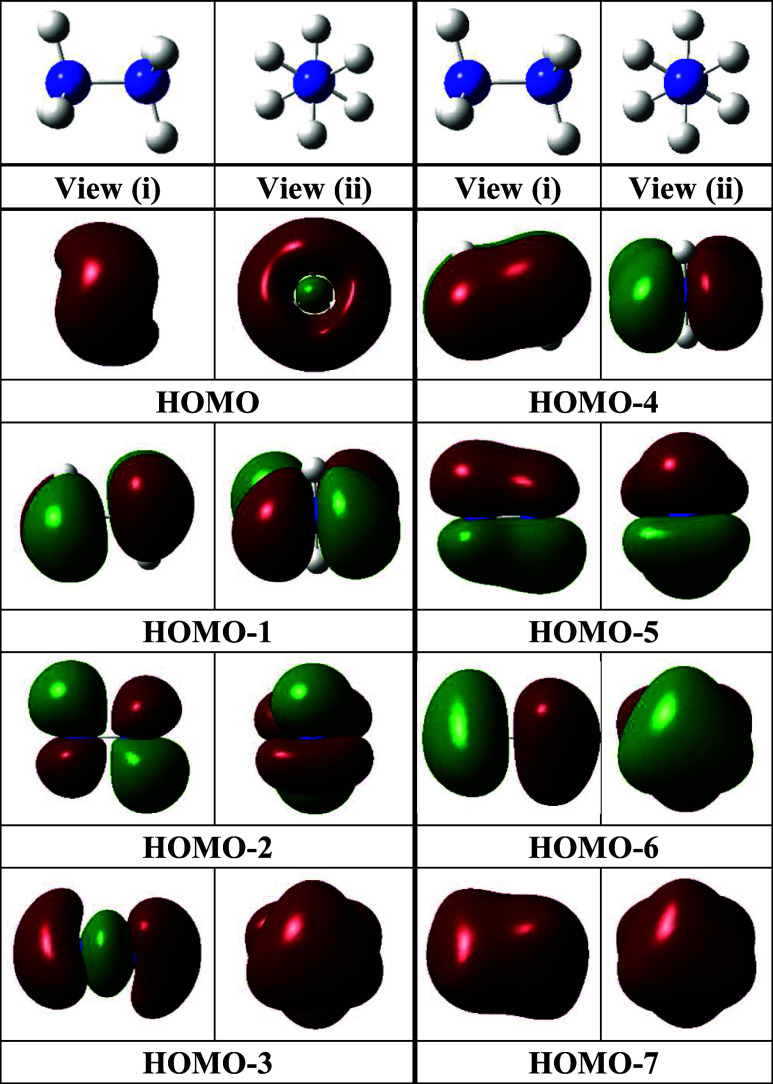
Images from two views of the highest occupied MOs for N_2_H_6_ at the ωB97XD/aug-cc-pVTZ level. Analogous pictures from CCSD­(T) calculations are in the SI.

The corresponding MOs obtained at the CCSD­(T) level are provided in the Supporting Information. The orbitals below the HOMO (i.e., HOMO–1 to HOMO–7) are analogous to those expected for ethane. The lowest (HOMO–7) is bonding across the whole molecule, and higher energy MOs are locally bonding within various regions of the NH_3_ fragments (HOMO–1, −2, −6), or across fragments (HOMO–4, −5). The HOMO–3 orbital is bonding with respect to the two N atoms. The large HOMO is distinct from any occupied orbital observed in ethane, however. It is fully bonding with respect to all six of the H atoms, but antibonding with respect to the six H–N bonds. From an NBO analysis (see the Supporting Information), diffuse Rydberg orbitals (3s and higher on N and *n*s orbitals on H) have a minor but not insignificant share (<1.5%) of the electron population. In N_2_H_6_, the HOMO arises it appears from a coupling of the lowest energy Rydberg orbitals with the N–H antibonding orbitals. We can report, however, that a stability analysis (a check on the wave function for external as well as internal instabilities) on the DFT (ωB97XD/aug-cc-pVTZ) optimized structure determined that the solution is stable. So, no complete active space (CAS) calculations have been undertaken in this work.

The bonding in N_2_H_6_ is not achieved by each NH_3_ lone pair establishing, for example, mutual pnictogen bonding type interactions with the adjacent N surface (as occurs in “double σ-hole” bonding between two polarized bases.^68^ It is also different from a Rydberg single bond as in ref 69. This N_2_H_6_ minimum on the (NH_3_)_2_ potential energy surface is achieved by a redistribution of the electron density of the NH_3_ molecules to generate an N–N bond (similar to that between the two C atoms in ethane (HOMO–3)), plus a multicenter Rydberg enhanced bond^70^ (HOMO) involving all of the H atoms. And the overall outcome of this bonding is a weakening of the N–H bonds accompanied by a planarization of the NH_3_ units, which suggests a strengthening of vicinal H---H interactions (but which may arise from a stabilization of the *D*_*3h*_ geometry of the NH_3_ fragments when Rydberg-like orbitals above the N lone pair *a*_1_ orbital are occupied)^71^ and, to a lesser extent, N–N bonding as well (Figure 2).

This observation is fully in line with a Wiberg bond order (obtained from a natural bond orbital (NBO) analysis) of 1.05 for N–N bonding at the ωB97XD/aug-cc-pVTZ level of theory—and 0.97 at the CCSD­(T)/aug-cc-pVTZ level, which cannot be accounted for by a simple N–N covalent bond. Such high bond orders (which, in the ωB97XD case, is in fact just above 1.0) is consistent with two weak N–N bonds (with an *average bond order* of ∼0.5/bond) though, in this N_2_H_6_ system, the lower energy N–N σ orbital (HOMO–3, Figure 2) is expected to contribute more to the bonding than the HOMO. This improbable N_2_H_6_ assembly, therefore—a remarkable minimum in the covalent region on the (NH_3_)_2_ dimer surface—exhibits double σ bonding between ammonia fragments.

Examining the electron distribution in the N_2_H_6_ system from another vantage point, we find that insights afforded by the so-called Adaptive Natural Density Partitioning (AdNDP) analysis of the Natural Bond Orbital picture, proves to be instructive. That treatment, which was carried out using the Multiwfn suite of programs,^63,64^ allows for a localized view of chemical bonding that goes nonetheless beyond the limited *2*-center *2*-electron description of chemical bonding to accommodate a more nuanced multicenter rendition of bonding where appropriate.

Restricting the lower bound of the orbital occupation to 1.90*e*, the eight “*n*-center, *n′*-electron” (*n*-c,*n′*-e) valence orbitals recovered may be described briefly as follows: one *2*-c,*2*-e N–N bond orbital, six *2*-c,*2*-e N–H bond orbitals, and one *8*-c,*2*-e orbital spanning the whole molecule and with a node between the outer H and central N atoms (Figure 3; more obvious in HOMO in Figure 2). That feature likely arises from the outer node of the N 3s orbital (which, as Pitzer pointed out,^72^ falls close to the H atoms in NH_3_) coinciding roughly with the nodes of the *a*_*1*_ LUMO of the NH_3_ molecule which is N–H antibonding but is H---H bonding between the two NH_3_ units as they meet in N_2_H_6_.

**Figure 3:**
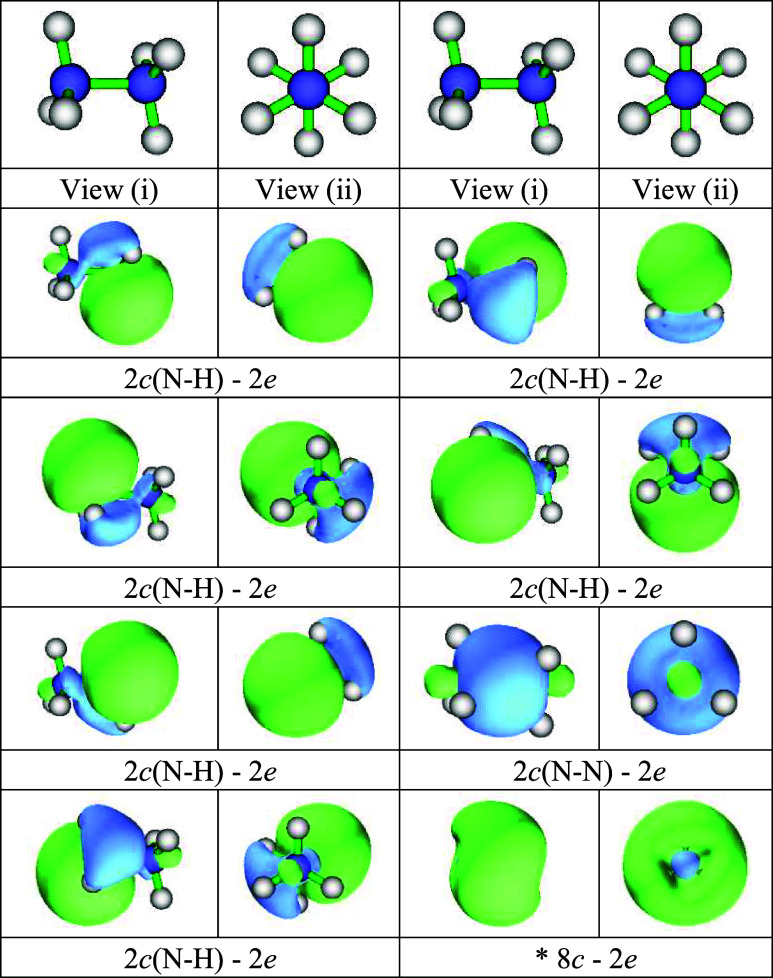
*n*-center *n*′-electron orbitals generated from an Adaptive Natural Density Partitioning (AdNDP) analysis of our NBO results. *This 8-center 2-electron orbital spans all of the atoms in molecule. It is bonding among the H atoms, and separately between the N centers as in the HOMO in the canonical molecular orbitals (Figure 2).

### Insights from Difference—C_2_H_6_^2–^ and N_2_H_6_

The ethane dianion (C_2_H_6_^2–^), as bleak a target as it is for experimental analysis, is isovalent with the neutral N_2_H_6_ unit. Indeed, the two species are formally isoelectronic, since C and N are in the same period (even the same (*p*) block), and the two species have the same structural arrangement and (*D*_*3d*_) symmetry.

Yet we find that those C_2_H_6_^2–^ and N_2_H_6_ species do not have quite the same electronic structure, a fact readily betrayed by structural data if we compare C_2_H_6_^2–^ and N_2_H_6_ with their less disconcerting 14-electron forms: ethane (C_2_H_6_) and the N_2_H_6_ dication (N_2_H_6_^2+^); see Figure 4.

**Figure 4:**
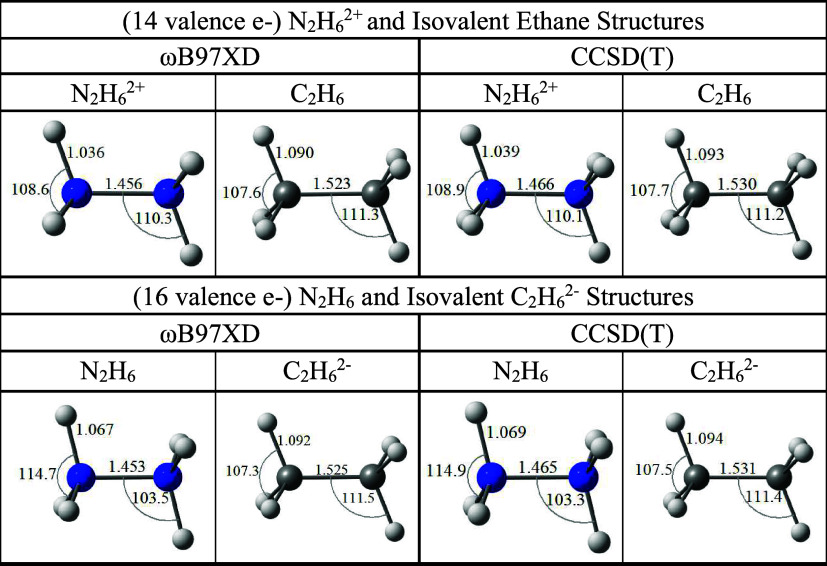
Minimum energy structures obtained using the ωB97XD and CCSD­(T) methods and aug-cc-pVTZ basis sets for N_2_H_6_^2+^ (top) and N_2_H_6_ (bottom) and their isovalent C analogues.

Adding two electrons to ethane in the gas phase is almost without structural consequence! The geometry is hardly affected at all (see the top and bottom ωB97XD and CCSD­(T) ethane structures in Figure 4). But that is not at all the case for the nitrogen analogues. In that case (Figure 4; going from N_2_H_6_^2+^ to N_2_H_6_), the geminal H atoms shift markedly away from each other, increasing H–N–H bond angles by 6°, while shrinking the H–N–N bond angle accordingly, and bringing the vicinal H atoms closer to each other. That structural modification, as well as the elongation of the N–H bonds is accompanied by an ever so slight N–N contraction. Indeed, the computed gas phase N–N distances in N_2_H_6_^2+^ (Figure 4) are already in range of the sum of two N radii (2 × 0.71 Å = 1.42 Å)^73^ even if they overshoot experimental values from extended solids (an average of 1.427(10) Å over ten different solids,^74^ and 1.434(8) Å in one other instance (ref 75). In that context, that even a tiny N–N contraction is achieved going from N_2_H_6_^2+^ to N_2_H_6_ is of note since it is in line with the occupation of a multicenter MO (the HOMO in Figure 2) that is bonding with respect to the vicinal H atoms, weakly bonding with respect to the N–N centers, and N–H antibonding. Since a similar structural reorganization is not observed for the C_2_H_6_/C_2_H_6_^2–^ pair, one anticipates, thanks to this structural evidence (Figure 4), that the MO that is occupied as we go from C_2_H_6_ to C_2_H_6_^2–^ is different in some way from the HOMO in Figure 2 that is occupied going from N_2_H_6_^2+^ to N_2_H_6_. And that is what we find (see HOMOs in Figure 5), but the situation is much subtler than it appears.

**Figure 5:**
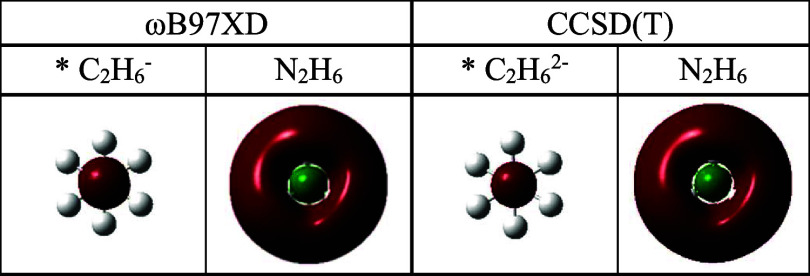
HOMOs of C_2_H_6_^2–^ and N_2_H_6_. * A coaxial portion of the C_2_H_6_^2–^ HOMO (out of phase with the (red) fragment on the C atoms in the first and third columns—surrounds that C–C bond, but its radial extent is such that is does not show up in the cube used to generate the HOMO on the selected (0.02 e·au^–3^) isosurface. A more complete picture (at 0.014 e·au^–3^) is provided in the SI.

Figure 5 inadvertently exaggerates the differences between the HOMOs of C_2_H_6_^2–^ and N_2_H_6_:; they are both expansive MOs with a core centered on the C or N centers (visible at the center in each image in Figure 5) with a coaxial outer shell (visible only for N_2_H_6_). That outer shell is missing for C_2_H_6_^2–^ in Figure 5, however, because the radial extent of that C_2_H_6_^2–^ MO is such that, at an isovalue of 0.02 e·au^–3^, which we use for all MOs herein, the outer regions of the MO are not captured in the default three-dimensional grid (cube). To capture a more complete picture of that C_2_H_6_^2–^ HOMO, we replotted it at a much smaller isovalue (see the SI), and found that the inner region of that HOMO, (visible in the first and third columns of Figure 5), is indeed enclose by a diffuse outer shell of opposite phase that is missing in Figure 5. So, a significant difference between the two HOMOs is in their spatial extents and not their basic form.

Why are these two “similar” systems so different? As an NBO analysis reveals, the bonding in C_2_H_6_^2–^ has a more substantial involvement of Rydberg orbitals (accounting for over 7% of the electron population compared to <2% for N_2_H_6_; see the SI), also, (i) unlike N_2_H_6_, C_2_H_6_^2–^ is an anion with a large charge imbalance, and (ii) since C has a lower electronegativity that N, it is not surprising that C_2_H_6_^2–^ does a poorer job stabilizing its HOMO. And all of those factors conspire to weaken the influence of the HOMO on structure and bonding (going from ethane to C_2_H_6_^2–^) compared to the isovalent N_2_H_6_^2+^/N_2_H_6_ case (Figure 5).

Following up on one reviewer’s suggestion, we reflect briefly here on links between the bonding in N_2_H_6_ and two other captivating dinitrogen hydrides. We pointed to the Rydberg bonded (NH_4_)_2_ species^33^ earlier in this work: that pair has a long contact between the NH_4_ fragments in a *D*_*3h*_ H-NH_3_···H_3_N–H arrangement with no direct N–N bond (see Figure S3). And its fascinating deprotonation product, the double Rydberg N_2_H_7_^–^ anion,^76–78^ features an asymmetric H bridge in a *C*_*3v*_ (H_3_N–H···NH_3_)^−^ complex (Figure S3). Both (NH_4_)_2_ and N_2_H_7_^–^lack the N–N bond characteristic of N_2_H_6_, and are evidently more dominated by Rydberg bonding compared to N_2_H_6_. The N_2_H_7_^–^ anion, for example, has a delocalized HOMO (Figure S3) that resides primarily on the periphery the three outer H atoms of the “H_3_N–H” fragment^77^ in the (H_3_N–H···NH_3_)^−^ arrangement, with a node between those H three atoms and that tetrasubstituted N center (Figure S3). That reminds us of nodal features observed in N_2_H_6_, but in that latter case, importantly, the nodes are associated with the H–N antibonding LUMO orbital of the NH_3_ units that play a decisive role in the symmetrical N_2_H_6_ structure.

### What of Analogous Non-Lewis Pairings?

We considered a number of RH_2_N-NH_2_R, and R_3_N-NR_3_ homodimer pairings of substituted N centers (for R = CH_3_, F, and Cl) as well as the heterodimers with NH_3_ to investigate the (non)-uniqueness of the observed binding of ammonia molecules. For all of those species, we specified starting (preoptimization) N–N distances comparable to the computed N_2_H_6_ value and a staggered orientation across the N-N bonds.

The outcomes: In some cases, the monomers simply parted ways and in other cases double elimination resulted in a substituted hydrazine (e.g., NH_3_ + NR_3_ → N_2_H_2_R_2_ + HR). Indeed, the specific outcome that a calculation yields—monomer separation or substituent dissociation with hydrazine formation—appears to depend in a complex way on the identity of the substituents, the level of theory, and the distance and orientation of the monomers relative to each other in the starting (preoptimization) structure.

We can report thus that we have not been able to locate any analogue of covalent N_2_H_6_ for any of the other pairs of systems we considered. In the absence of a thorough study of the potential energy surfaces of those and other homo- and heterodimers, however, we cannot conclude that analogous non-Lewis minima do not dent the surfaces of substituted amine dimers (or period 2 pairs more broadly), but at this point, we are unaware of any other such instance.

### The Covalent Minimum and Competing Channels

The covalent N_2_H_6_ minimum has been identified in this work on the potential energy surface for several different levels of theory. But what is the nature of the potential energy surface in the vicinity of that minimum? What is the depth of the potential well? What of any competing channel leading to lower energy products or isomers of the ammonia pair?: here we are thinking in particular about hydrazine formation (i.e., the elimination of two H atoms, with possible H_2_ formation—see ref 29) or a collapsed to the weakly bound and well-studied H_3_N---HNH_2_ ammonia dimer.^31,32^

To probe that question, we carried out a relaxed potential energy scan at the ωB97XD/aug-cc-pVTZ level starting from a compressed version of the N_2_H_6_ minimum with an N–N separation of 1.140 Å and increasing by 0.01 Å to a point (2.60 Å) well beyond the N_2_H_6_ minimum (1.453 Å). Beyond the controlled elongation of the N–N distance, no constraint was imposed on the structure during the scan. Unsurprisingly at this point in our discussion, the analysis exposed a local minimum consistent with the covalent N_2_H_6_ structure (Figure 6).

**Figure 6:**
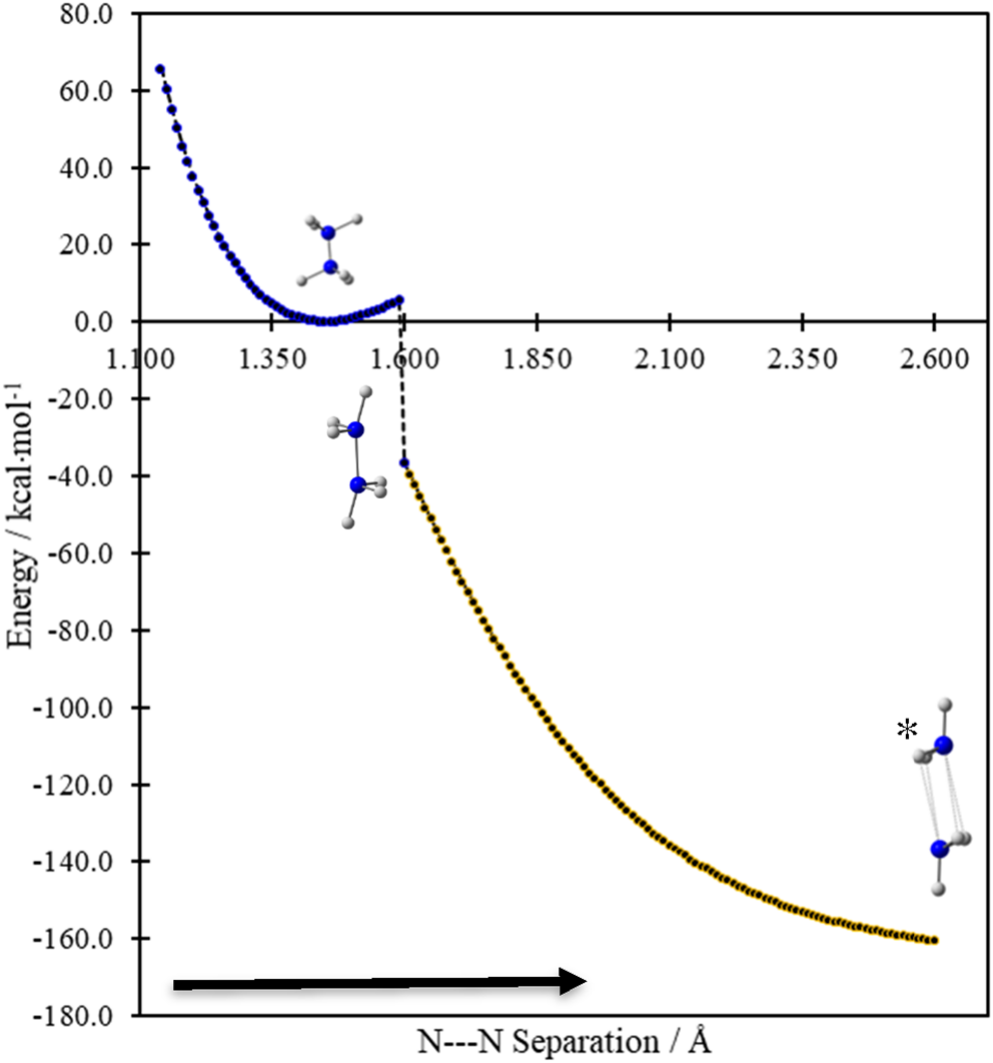
Outcome of a relaxed potential energy scan obtained by increasing the N---N distance by 0.01 Å increments from a compressed N_2_H_6_ structure (N–N distance = 1.14 Å) to a separation of 2.6 Å. At about 1.60 Å, the otherwise unconstrained structure hops through an internal conversion to a lower energy curve where the lone pair on each monomer is more accessible to H atoms of the adjacent NH_3_ fragment. *The lower curve leads to a weak dimer arrangement at the end of the scan that is optimized subsequently to the H-bonded dimer shown in Figure 7.

Beyond 1.590 Å during the scan, however, (at a well depth of ∼5.5 kcal·mol^–1^ beyond the N_2_H_6_ minimum) a hop or discontinuous collapse to a lower energy though more loosely bound dimer structure is observed. That drastic transformation allows for the direct exposure of the H atoms of each monomer to the lone pair on the adjacent N center (Figures 6 and 7).

**Figure 7:**
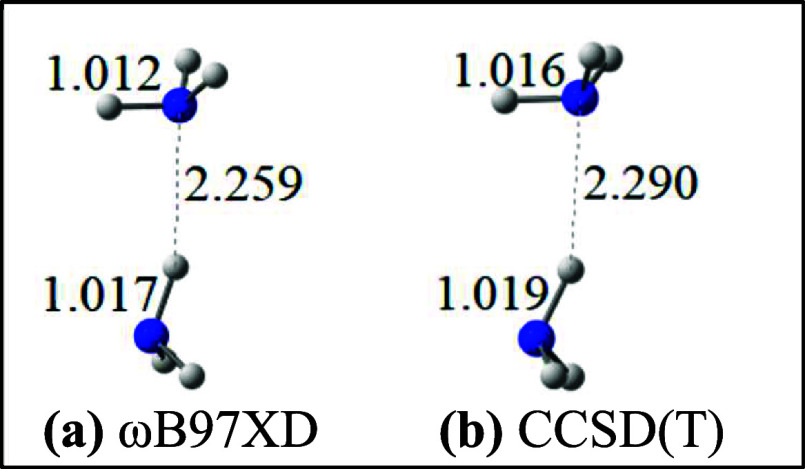
Minimum energy structures obtained using the ωB97XD and CCSD­(T) methods using the aug-cc-pVTZ basis sets for a weak hydrogen bonded ammonia dimer.

This N_2_H_6_ structure is fragile, and is threatened from all directions. Stretching the N–N bond a few tenths of an angstrom beyond the N_2_H_6_ minimum leads to a collapse to a weak hydrogen bonded dimer, and dissociation to H_3‑n_NNH_3‑n_ and nH_2_ is likely at very short distances. So, a route to experimental realization starting from two NH_3_ monomers is fraught with obstacles.

Moreover, we should point out that the well depth achieved in Figure 6 prior to the rearrangement for weak dimer formation (Figure 7) is much lower than the computed zero-point energy for N_2_H_6_ (>40.0 kcal·mol^–1^; Figure 1). So, pessimism about the possible preparation of such a structure is highly justifiable. The exceedingly rich value of this system lies, however, in what it exposes about the remarkable possibilities for chemical bonding under unconstrained conditions (without spatial confinement or high pressure, for instance), as well as the nature and limits of the partnership between traditional covalent and Rydberg bonding in N_2_H_6_.

Instead of two ammonia molecules being coaxed to form N_2_H_6_, might N_2_H_6_ be approached from other directions? Several compounds containing N_2_H_6_^2+^ (hydrazinediium or hydrazinium­(2+)) dication are known.^75,79^ We have been unable to find any report on efforts to prepare N_2_H_6_ from the closed shell dication N_2_H_6_^2+^ or from N_2_H_4_—safety hazard that the latter is—through strategies akin to those deployed for NH_4_ formation from NH_3_,^80^ for example. The prospects are as intriguing as the bonding in the N_2_H_6_ system, which is evidently unique among the period 2 *p*-block hydrides despite its high energy and apparently low barrier to falling apart.

## Conclusions

The chemical bonding, energetics, and nature of the potential energy surface of the covalently bound hexahydridodinitrogen (N_2_H_6_) minimum energy species on the (NH_3_)_2_ potential energy surface of the ammonia dimer have been examined. The structure in question has a remarkably short N–N bond, with the three terminal hydrogens on each N center arranged in a staggered *D*_*3d*_ arrangement akin to the structure of ethane. Unlike two ·CH_3_ radical fragments, however, which are expected to be stabilized through the formation of the ethane molecule, the ammonia fragments of the N_2_H_6_ (H_3_N-NH_3_) species—each with a complete octet (hence no unpaired electron)—are stable isolated molecules on their own. So, the basis for the N_2_H_6_ minimum,^29^ which runs counter to conventional expectations, is not obvious.

The electron distribution in that species is interpreted based on a molecular orbital theoretical perspective as a double σ bond: a two-center two-electron N–N bond, plus a multicenter two-electron bond that is antibonding with respect to the N–H bonds, but bonding among the H centers and (to a weaker extent) the N centers as well. Additional support for that picture is provided herein. For very short N–N distances (on the repulsive branch of the H_3_N-NH_3_ potential energy curve) the release of two H atoms with hydrazine (N_2_H_4_) formation is favored and for long N---N distances (on the attractive branch) the formation of a hydrogen bonded dimer (H_3_N···H-NH_2_) is a competing channel as well on the ammonia dimer surface. N_2_H_6_ is very high in energy compared to two isolated ammonia molecules as well as the weak hydrogen bonded dimer. Barriers to any eventual preparation of N_2_H_6_ are clear. Yet, the system is captivating for the insight it provides in its extraordinary structural and electronic profile, and in what it represents as an inspiration to continue to push against perceived boundaries in chemical bonding.

## Supplementary Material





## Notes

Molecular orbitals obtained at other levels of theory, HOMO–LUMO gaps, and NBO data for key systems (PDF)
A set of “.xyz” files with viewable Cartesian coordinates for systems optimized in this work (ZIP)
