# Supplementary material for: On Covalent N2H6: A High-Energy, Non-Lewis, Local Minimum That May (Not) Exist
Source: J Phys Chem A. 2026 Apr 6;130(15):3048–57. doi: 10.1021/acs.jpca.6c01311 (PMC13093481; doi:10.1021/acs.jpca.6c01311)
Supplement: Supplementary file 1 [file jp6c01311_si_001.pdf]

On Covalent N_2H_6: A High-Energy, Non-Lewis, Local Minimum that May (Not) Exist

Kelling J. Donald* and George Ruthven

Department of Chemistry, Gottwald Center for the Sciences, University of Richmond, Richmond, Virginia 23173, United States

Supporting Information

* Corresponding author. K. J. Donald, Tel.: 1-804-484-1628. E-mail: kdonald@richmond.edu ORCID: 0000-0001-9032-4225

Table of Contents

Abbreviated Captions*	Page
Guide to *.xyz* Files	S3
**Table S1**: Data showing the basis set dependence of N_2H_6 optimization using the HF method.	S3
**Table S2**: NBO data for N_2H_6: ωB97XD and CCSD(T) methods and aug-cc-pVTZ basis set.	S4
**Table S3**: Frontier orbital energies, and HOMO-LUMO Gaps for N_2H_6 and $C_2H_6^{2-}$	S5
**Figure S1**: Images of MOs for N_2H_6 obtained at the CCSD(T)/aug-cc-pVTZ level.	S6
**Figure S2**: HOMO of $C_2H_6^{2-}$ at the ωB97XD/aug-cc-pVTZ level, at two different isovalues: 0.02 and the much lower 0.0053 isovalue, the radial extent of the outer region of that $C_2H_6^{2-}$ orbital is such that – typical of Rydberg orbitals – it engulfs the small molecule.	S7
**Figure S3:** Computed ωB97XD/aug-cc-pVTZ structures and HOMOs (in two views) for N_2H_6, $N_2H_7^-$, and $(NH_4)_2$**.**	S8

*More detailed captions are provided with the individuals figures and tables.

Guide to .xyz files

A set of *.xyz* files containing the optimized coordinates for systems considered in this work is included as part of the supporting information.

TABLES

Table S1: Data showing the basis set dependence of N_2H_6 optimization using the HF method.

	6-311+G*	6-311++G**	def2-TZVPP	def2-QZVPP	cc-pVDZ	cc-pVTZ	cc-pVQZ	cc-pV5Z
	**Bond Distances / Å**							
N-N	1.415	1.425	1.428	1.427	1.436	1.430	1.427	1.425
N-H	1.026	1.036	1.045	1.041	1.072	1.048	1.041	1.039
Angles	**Angles / º**							
N-N-H	106.5	106.9	104.7	105.5	101.8	104.4	105.2	105.8
H-N-H	112.3	111.9	113.8	113.1	115.9	114.1	113.4	112.8

Table S2: Natural Bond Orbital (NBO) data for N_2H_6 and $C_2H_6^{2-}$ structures optimized using the ωB97XD and CCSD(T) methods and aug-cc-pVTZ basis sets.

N_2H_6 (ωB97XD)

```
                         Natural Population
-------------------------------------------------------------------
  Core                          3.99940 ( 99.9850% of   4)
  Valence                      15.75235 ( 98.4522% of  16)
  Natural Minimal Basis        19.75175 ( 98.7588% of  20)
  Natural Rydberg Basis         0.24825 (  1.2412% of  20)
-------------------------------------------------------------------

   Atom  No          Natural Electron Configuration
----------------------------------------------------------------------------
     N    1   [core]2S( 1.65)2p( 4.27)3S( 0.01)3d( 0.02)4p( 0.02)
     H    2            1S( 0.65)2p( 0.01)4S( 0.01)
     H    3            1S( 0.65)2p( 0.01)4S( 0.01)
     H    4            1S( 0.65)2p( 0.01)4S( 0.01)
     N    5   [core]2S( 1.65)2p( 4.27)3S( 0.01)3d( 0.02)4p( 0.02)
     H    6            1S( 0.65)2p( 0.01)4S( 0.01)
     H    7            1S( 0.65)2p( 0.01)4S( 0.01)
     H    8            1S( 0.65)2p( 0.01)4S( 0.01)

Wiberg bond index, Totals by atom:

    Atom    n
    ---------------
  1.  N  2.7688
  2.  H  0.9221
  3.  H  0.9221
  4.  H  0.9221
  5.  N  2.7688
  6.  H  0.9221
  7.  H  0.9221
  8.  H  0.9221
```

$C_2H_6^{2-}$ (ωB97XD)

```
                         Natural Population
-----------------------------------------------------------------
  Core                          3.99915 ( 99.9788% of   4)
  Valence                      14.52747 ( 90.7967% of  16)
  Natural Minimal Basis        18.52662 ( 92.6331% of  20)
  Natural Rydberg Basis         1.47338 (  7.3669% of  20)
-----------------------------------------------------------------

   Atom  No          Natural Electron Configuration
-----------------------------------------------------------------
     C    1   [core]2S( 1.17)2p( 3.51)4S( 0.03)3d( 0.01)
     H    2            1S( 0.86)2S( 0.22)
     H    3            1S( 0.86)2S( 0.22)
     H    4            1S( 0.86)2S( 0.22)
     C    5   [core]2S( 1.17)2p( 3.51)4S( 0.03)3d( 0.01)
     H    6            1S( 0.86)2S( 0.22)
     H    7            1S( 0.86)2S( 0.22)
     H    8            1S( 0.86)2S( 0.22)

Wiberg bond index, Totals by atom:

    Atom    n
    ---------------
  1.  C  3.9140
  2.  H  1.3839
  3.  H  1.3832
  4.  H  1.3841
  5.  C  3.9140
  6.  H  1.3841
  7.  H  1.3838
  8.  H  1.3832
```

N_2H_6 (CCSD(T))

```
                         Natural Population
--------------------------------------------------------------------
  Core                          3.99944 ( 99.9859% of   4)
  Valence                      15.71421 ( 98.2138% of  16)
  Natural Minimal Basis        19.71365 ( 98.5682% of  20)
  Natural Rydberg Basis         0.28635 (  1.4318% of  20)
--------------------------------------------------------------------

   Atom  No          Natural Electron Configuration
----------------------------------------------------------------------------
     N    1  [core]2S( 1.68)2p( 4.34)3S( 0.01)3p( 0.02)3d( 0.03)
     H    2            1S( 0.61)2p( 0.01)4S( 0.02)
     H    3            1S( 0.61)2p( 0.01)3S( 0.02)
     H    4            1S( 0.61)2p( 0.01)3S( 0.02)
     N    5   [core]2S( 1.68)2p( 4.34)3S( 0.01)3p( 0.02)3d( 0.03)
     H    6            1S( 0.61)2p( 0.01)4S( 0.02)
     H    7            1S( 0.61)2p( 0.01)3S( 0.02)
     H    8            1S( 0.61)2p( 0.01)4S( 0.02)

Wiberg bond index, Totals by atom:

    Atom    n
    ----------------
  1.  N  2.6142
  2.  H  0.8949
  3.  H  0.8961
  4.  H  0.8971
  5.  N  2.6165
  6.  H  0.8966
  7.  H  0.8954
  8.  H  0.8943
```

$C_2H_6^{2-}$ (CCSD(T))

```
                         Natural Population
--------------------------------------------------------------------
  Core                          3.99918 ( 99.9796% of   4)
  Valence                      14.25489 ( 89.0931% of  16)
  Natural Minimal Basis        18.25407 ( 91.2704% of  20)
  Natural Rydberg Basis         1.74593 (  8.7296% of  20)
--------------------------------------------------------------------

   Atom  No          Natural Electron Configuration
------------------------------------------------------------------
     C    1   [core]2S( 1.13)2p( 3.41)4S( 0.03)4d( 0.01)
     H    2            1S( 0.86)2S( 0.27)
     H    3            1S( 0.86)2S( 0.27)
     H    4            1S( 0.86)2S( 0.27)
     C    5   [core]2S( 1.13)2p( 3.41)4S( 0.03)4d( 0.01)
     H    6            1S( 0.86)2S( 0.27)
     H    7            1S( 0.86)2S( 0.27)
     H    8            1S( 0.86)2S( 0.27)

Wiberg bond index, Totals by atom:

    Atom    n
    --------------
  1.  C  3.9683
  2.  H  1.4536
  3.  H  1.4538
  4.  H  1.4534
  5.  C  3.9681
  6.  H  1.4527
  7.  H  1.4561
  8.  H  1.4534
```

Table S3: Frontier orbital energies (in Hartree units), and HOMO-LUMO Gaps for N_2H_6 and $C_2H_6^{2-}$ using the aug-cc-pVTZ basis set.

	**ωB97XD**		**CCSD(T)**	
	**N_2H_6**	**$C_2H_6^{2-}$**	**N_2H_6**	**$C_2H_6^{2-}$**
**HOMO-1**	-0.62589	-0.17900	-0.72394	-0.24485
**HOMO**	-0.18112	0.11945	-0.15965	0.13584
**LUMO**	0.00557	0.19884	0.01641	0.21240
**ΔE_{H-L} / Hartrees**	**0.1867**	**0.0794**	**0.1761**	**0.0766**
**ΔE_{H-L} / kcal·mol^{-1}**	**117.1**	**49.8**	**110.5**	**48.0**
**ΔE_{H-L} / eV**	**5.08**	**2.16**	**4.79**	**2.08**

FIGURES

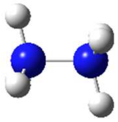	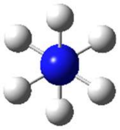
Side View (i)	Side View (ii)
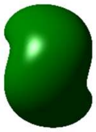	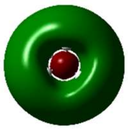
HOMO	HOMO
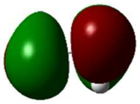	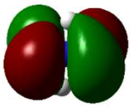
HOMO-1	HOMO-1
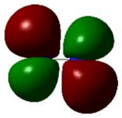	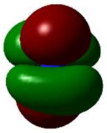
HOMO-2	HOMO-2
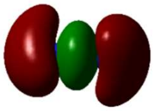	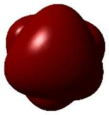
HOMO-3	HOMO-3
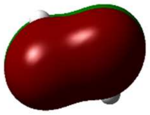	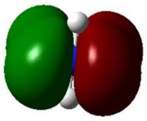
HOMO-4	HOMO-4
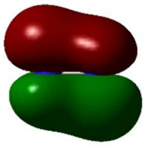	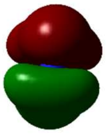
HOMO-5	HOMO-5
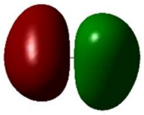	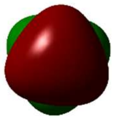
HOMO-6	HOMO-6
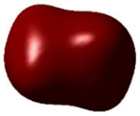	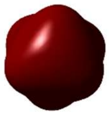
HOMO-7	HOMO-7

Figure S1: *Images from two views of the highest occupied MOs for N_2H_6 at the CCSD(T)/aug-cc-pVTZ level.*

Isovalue / e·au^{-3}	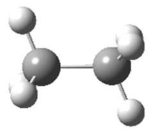	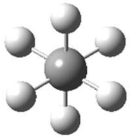
	HOMO Side View (i)	HOMO Side View (ii)
0.02	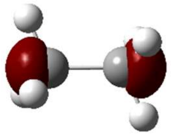	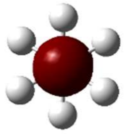
0.0053	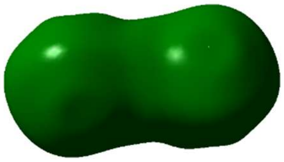	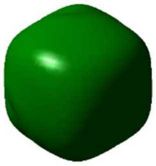

Figure S2: *Images, from two views, of the highest occupied MO for $C_2H_6^{2-}$ at the ωB97XD/aug-cc-pVTZ level at two different isovalues, in e·au^{-3}. At the much lower 0.0053 isovalue, the radial extent of the outer region of that $C_2H_6^{2-}$ orbital is such that – typical of Rydberg orbitals – it engulfs the small molecule. The outer region (green) of the MO encapsulates, across a radial node, the localized inner (red) region on the C centers. That core is visible at the 0.02 isovalue above (red) because the outer region is quite diffuse and falls outside the cube from which that 0.02 MO image is plotted.*

<table>
<tr><th></th><th>N_2H_6</th><th>$N_2H_7^- \equiv (H_3N\text{-}H\cdots NH_3)^-$</th><th>$(NH_4)_2$</th></tr>
<tr><td rowspan="2">Structure</td><td>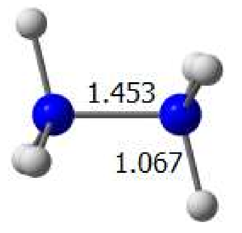
</td><td>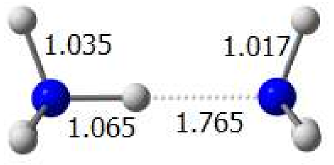
</td><td>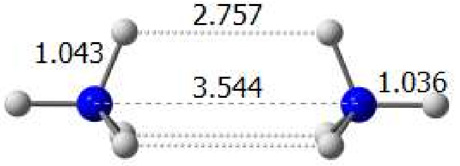
</td></tr>
<tr><td>D_{3d}</td><td>C_{3v}</td><td>D_{3h}</td></tr>
<tr><td>HOMO (view 1)[a]</td><td>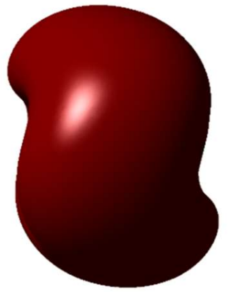</td><td>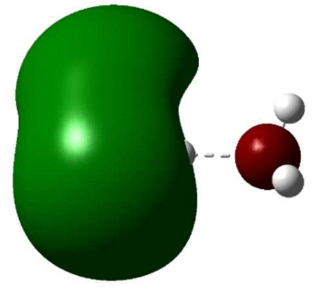</td><td>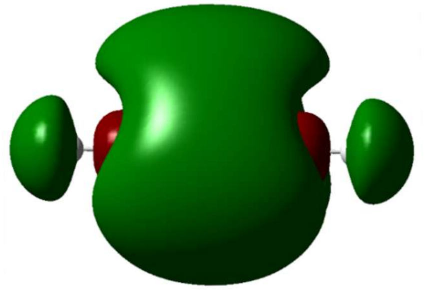</td></tr>
<tr><td>HOMO (view 2)[b]</td><td>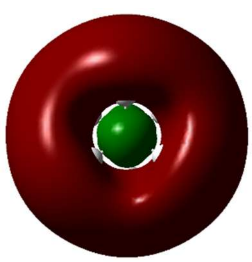</td><td>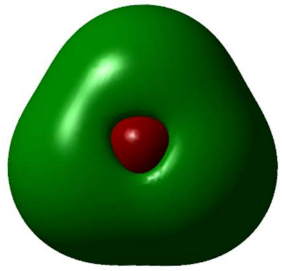</td><td>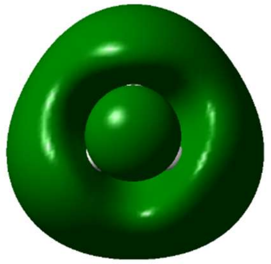</td></tr>
<tr><td>HOMO (view 3)[c]</td><td>-</td><td>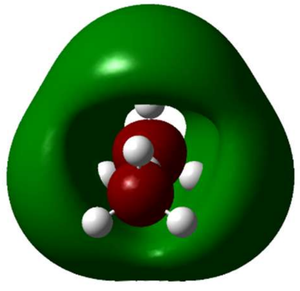</td><td>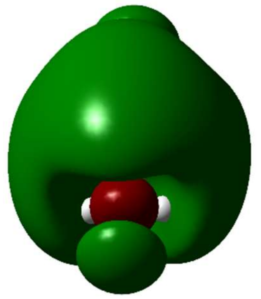</td></tr>
</table>

Figure S3: *Computed ωB97XD/aug-cc-pVTZ structures and HOMOs for N_2H_6, $N_2H_7^-$, and $(NH_4)_2$. Despite key differences in the bonding, all three HOMOs exhibit a substantial delocalization about the whole molecule. The HOMOs were generated for an isovalue of 0.0218 $e{\cdot}au^{-3}$. [a]View 1 is a side view of their HOMOs, with each molecule oriented precisely as it is shown in the 'Structure' row above it in this figure. [b]View 2 shows the HOMOs as they would appear to a viewer looking at them, still in the view 1 orientation, but from the left (in the plane of the page). [c]View 3 is a tilted orientation of the Rydberg HOMOs of $N_2H_7^-$ and $(NH_4)_2$, offering additional insight into the spatial expanse of those orbitals.*

NOTE: *View 3 is especially useful for seeing the asymmetric HOMO of $N_2H_7^-$ from the side opposite view 2. For the $N_2H_7^-$ and $(NH_4)_2$ HOMOs, the electron density lies largely out on the periphery of the H atoms, and mostly around the tetrahedral 'H_3N-H' fragment in the case of $N_2H_7^-$.*
